# Perioperative hemodynamic optimization using the photoplethysmography in colorectal surgery (the PANEX3 trial): study protocol for a randomized controlled trial

**DOI:** 10.1186/s13063-016-1278-4

**Published:** 2016-03-22

**Authors:** Marc-Olivier Fischer, Anne-Lise Fiant, Mariam Boutros, Frédéric Flais, Tzetan Filipov, Stéphane Debroczi, Léa Pasqualini, Toufiq Rhanem, Jean-Louis Gérard, Lydia Guittet, Jean-Luc Hanouz, Arnaud Alves, Jean-Jacques Parienti, For the PANEX3 study group

**Affiliations:** Pôle Réanimations Anesthésie SAMU/SMUR, CHU de Caen, Avenue de la Côte de Nacre, CS 30001, F-14000 Caen, France; EA 4650, Université de Caen Basse-Normandie, Esplanade de la Paix, CS 14 032, F-14000 Caen, France; Department of Public Health, CHU de Caen, Avenue de la Côte de Nacre, CS 30001, F-14000 Caen, France; INSERM1086, Faculty of Medicine, Caen University Hospital, Avenue de la Côte de Nacre, F-14032 Caen, Cedex France; Service de chirurgie digestive, CHU de Caen, Avenue de la Côte de Nacre, CS 30001, F-14000 Caen, France; Department of Biostatistics and Clinical Research, CHU de Caen, Avenue de la Côte de Nacre, CS 30001, F-14000 Caen, France

**Keywords:** Anesthesiology, Hemodynamic, Abdominal surgery, Plethysmography

## Abstract

**Background:**

Photoplethysmography with a digital sensor (ClearSight, Edwards Lifesciences, Irvine, CA, USA) connected to a dedicated monitor (EV 1000, Edwards Lifesciences) was recently proposed for use in performing hemodynamic optimization during surgery. The objective of this study is to evaluate the effect of photoplethysmography on the incidence of postoperative complications compared with the conventional hemodynamic algorithm, which uses mean arterial pressure.

**Methods/design:**

The hemodynamic optimization using photoplethysmography (PANEX3) trial is a monocentric, randomized, single-blind, controlled, two parallel arm, superiority trial, randomizing 160 patients with an intermediate risk of postoperative complications after colorectal surgery. Informed consent will be obtained from all participants. The hemodynamic optimization is conducted using a specified hemodynamic algorithm either with photoplethysmography (the photoplethysmography group) or with conventional mean arterial pressure (the control group). The anesthesiologist performed a 1:1 randomization the day before surgery using a scratch card, which is available 24/7. The randomization sequence is generated using permutated blocks. Both the patients and surgeons are blinded to the allocation group. The primary outcome is the incidence of at least one postoperative complication during the 30 days following surgery. Two independent experts, who were blinded to the group allocations, validate the complication for each patient using an a priori classification. The secondary outcomes are to study the total number of postoperative complications, the real length of hospital stays, and the postoperative mortality between each group.

**Discussion:**

The PANEX3 trial is the first randomized controlled study conducted to investigate whether perioperative hemodynamic optimization using photoplethysmography during colorectal surgery could decrease the incidence of patients having at least one postoperative complication.

**Trial Registration:**

ClinicalTrials.gov Identifier: NCT02343601

## Background

Although more than 320 million surgeries are performed worldwide each year [1], recent data show that the perioperative morbidity and mortality remain significant [2]. The concept of perioperative hemodynamic optimization is important to decrease the morbidity and length of the hospital stay after noncardiac surgery [3]. Currently, expert guidelines recommend a maximization of stroke volume using titrated fluid loading with cardiac output monitoring [4, 5]. However, although such a strategy has been shown to be beneficial for patients, it is rarely used at the bedside [6, 7]. The invasiveness of cardiac output monitoring, lack of knowledge, and time constraints could partially explain these disappointing results [8]. Noninvasive and plug-and-play cardiac output monitoring could increase the adherence to the guidelines. Using a simple finger sensor, the plethysmography displays continuous blood pressure and beat-to-beat cardiac output measurements. Previous studies reported encouraging results with this technology during the perioperative period [8–11]. However, no phase III study has been conducted to assess the clinical utility of photoplethysmography to decrease the perioperative morbidity [12], and a benefit remains to be established.

The PANEX3 study aims to compare in patients with an intermediate risk of postoperative complications after colorectal surgery the effects of photoplethysmography using a perioperative hemodynamic optimization algorithm with a conventional hemodynamic algorithm that uses mean arterial pressure.

## Methods/design

### Ethics and study design

**Fig. 1:**
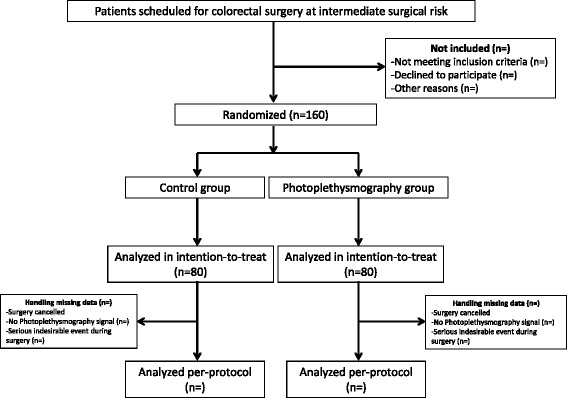
Consort diagram of the PANEX3 trial

The perioperative hemodynamic optimization using photoplethysmography in colorectal surgery (PANEX3) study is a randomized, controlled, two-arm trial. The institutional review board (IRB) of the University Hospital of Caen (Comité de Protection des Personnes Nord-Ouest III, Avenue la Côte de Nacre, Niveau 03, Porte 03–508, 14 033 Caen Cedex 9, France) approved the study (Registration number ID RDB: 2014-A00870-47 on 3 December 2014). The PANEX3 study is being conducted in accordance with the Declaration of Helsinki and was registered on 13 June 2014 on the ClinicalTrials.gov website with the trial identification number NCT02343601. The PANEX3 trial follows the CONSORT statement [13], and the CONSORT diagram is given in Fig. 1. All patients are asked for written informed consent, as required by the IRB, in accordance with the Declaration of Helsinki; informed consent will be obtained from all participants.

### Study population

All patients for planned colorectal surgeries are eligible for the study.

Patients fulfilling one or more of the following criteria will not be included: lack of informed consent prior to randomization, use of another hemodynamic monitor for cardiac output measurement, chronic kidney disease with creatinine clearance < 30 ml/min, black skin (limit of photoplethysmography technology), pregnant patients, age < 18 years, or under legal protection. This provides good technical conditions for the photoplethysmography and a relatively homogenous study population for the interpretation of the results.

The included patients are at moderate surgical risk, based on the surgical procedure and/or the medical history for each patient. Patients with high surgical risk according to the medical history will have a cardiac output monitor [4, 5], and they will be not included in the study.

### Randomization

Randomization is performed by the anesthesiologist the day before surgery using a scratch card available 24/7 in the hospital. The randomization sequence is generated using permutated blocks.

### Interventions

On arrival in the operating room, each patient receives the usual monitoring, including a lead ECG, noninvasive blood pressure, pulse oximetry, and a photoplethysmography monitor using a digital sensor (ClearSight, Edwards Lifesciences) connected to a dedicated monitor (EV 1000, Edwards Lifesciences). Included patients are assigned to the control group (blinded monitor) or the photoplethysmography group (monitor available to guide anesthesia), according to the randomization. After the heart reference system is zeroed at the mid-axillary line, the monitoring is continuously recorded from the start of general anesthesia induction until discharge from the recovery room. All photoplethysmography data is then recovered by an independent investigator not involved in the patient care.

**Fig. 2:**
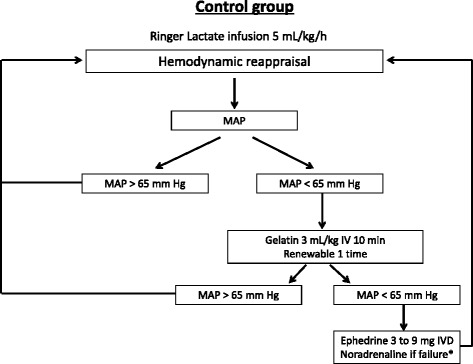
Hemodynamic algorithm for the control group. *Norepinephrine after the failure of the use of ephedrine, which is defined by the use of 30 mg of ephedrine without the desired hemodynamic response. Norepinephrine: dosage began at 0.05 μg/kg/min and then was adjusted in steps of 0.05 μg/kg/min. MAP, mean arterial pressure

In the control group (Fig. 2), the hemodynamic goal is a mean arterial pressure (MAP) > 65 mmHg; if the MAP is under this value, the clinician can prescribe an intravenous fluid challenge using 3 ml/kg of gelatin over 10 minutes, renewable once, and then a vasopressor thereafter (ephedrine until 30 mg, and norepinephrine thereafter).

**Fig. 3:**
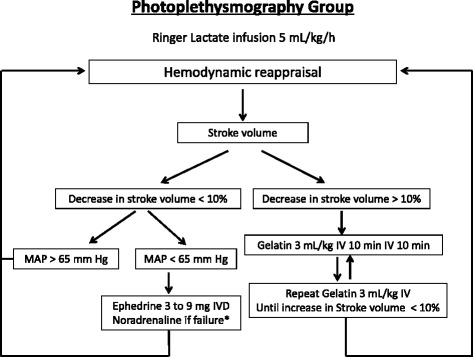
Hemodynamic algorithm for the photoplethysmography group. *Norepinephrine after the failure of the use of ephedrine, which is defined by the use of 30 mg of ephedrine without the desired hemodynamic response. Norepinephrine: dosage began at 0.05 μg/kg/min and then was adjusted in steps of 0.05 μg/kg/min. MAP, mean arterial pressure

In the photoplethysmography group (Fig. 3), the hemodynamic target includes the stroke volume and the MAP: the fluid challenge prescription depends on the changes in stroke volume, according to recent guidelines [4], whereas the vasopressor use depends on a MAP < 65 mmHg after the maximization of the stroke volume.

The allocated therapy is delivered until discharge from the recovery room.

### Standard procedures

The anesthesia medications follow the local protocol for the induction (propofol, sufentanil, and atracurium) and the maintenance of anesthesia (sevoflurane, sufentanil, and atracurium). The postoperative pain management follows another local protocol using intravenous (IV) lidocaine (1.5 mg/kg intravenous drip (IVD) at the start of anesthesia, then 2 mg/kg/h in a syringe driver during surgery, and 1.5 mg/kg/h during recovery room), paracetamol (1 g x 4 per day), nefopam (80 to 100 mg in syringe driver per day), and oxycodone (5 mg up to 4 per day if pain evaluation > 3/10). For all patients, surgeons perform scar infiltration with 20 ml of ropivacaine 0.75 %. The concomitant use of regional anesthesia (transverse abdominal plane block with 20 ml of ropivacaine 0.75 %, and/or spinal anesthesia using morphine 150 to 400 μg) is left to the discretion to the attending anesthesiologist.

The antibiotic prophylaxis and enhanced recovery after the elective colorectal surgery program follow the recent guidelines [14].

The ventilator settings include a tidal volume using 6 to 8 ml/ideal body weight, a positive end-expiratory pressure between 5 and 8 cm H_2_O, lung recruitment maneuvers (30 cm H_2_O during 30 seconds every 30–45 minutes), a respiratory rate and an FiO_2_ according to a range of end-tidal carbon dioxide tension between 35 and 45 mm Hg, and a pulse oximetry > 96 %.

### Study endpoints

The primary outcome is the incidence of at least one postoperative complication during the 30 days following surgery. Two independent experts confirm the presence of the complication using an a priori classification (Table 1) in accordance with recent guidelines [15]. These experts are not involved in the study and work in a different university hospital.

**Table 1:** A priori definition of postoperative complications

Complication	Definition
Paralytic ileus	Failure to tolerate solid food or defecate for 3 or more days after surgery
Infection, source uncertain	Strong clinical suspicion of infection, but the source has not been confirmed because clinical information suggests more than one possible site, meeting two or more of the following criteria: core temperature < 36 °C or > 38 °C, white cell count > 12 x 10^9^/l or < 4 x 10^9^/l, respiratory rate > 20 breath/minute or PaCO_2_ < 4.7 kPa, pulse rate > 90/min.
Surgical site infection (superficial)	Infection occurs within 30 days after surgery and involves only skin and subcutaneous tissue of the incision and (purulent drainage from superficial incision, organisms isolated from superficial surgical site, or diagnosis of incisional surgical site infection by a surgeon or attending physician, or clinical symptoms in this surgical superficial site.
Surgical site infection (deep)	Infection occurs within 30 days after surgery and involves deep soft tissues and purulent drainage, or abscess or other evidence of infection during surgery or radiological examination, or a deep incision spontaneously dehisces or is deliberately opened by a surgeon and its culture is positive or the patient is symptomatic.
Surgical site infection (organ/space)	Infection that involves any part of the body, excluding the fascia or muscle layers, and meets the criteria further indicated. Infection occurs within 30 days after surgery, and the infection appears to be related to the surgical procedure and involves any part of the body, excluding the skin incision, fascia or muscle layers opened or manipulated during the operative procedure, and the patient has at least one of the following criteria: purulent drainage from a drain that is placed through a stab wound into the organ/space; or organisms isolated from an aseptically obtained culture of fluid or tissue in the organ/space; or an abscess or other evidence of infection involving the organ/space that is found on direct examination, during reoperation, or by histopathological or radiological examination; or diagnosis of an organ/space surgical site infection by a surgeon or attending physician).
Laboratory-confirmed bloodstream infection	Patient has a recognized pathogen cultured from one or more blood cultures, and the organism cultured from the blood is not related to an infection at another site, or the patient had clinical signs (fever > 38 °C, hypotension, or chills) and at least one of the following common skin contaminants cultured from two or more blood cultures drawn on separate occasions, or one blood culture from a patient with an intravascular line, and the physician institutes appropriate antimicrobial therapy, or positive blood antigen test.
Anastomotic breakdown	Leak of luminal contents from a surgical connection between two hollow viscera. The luminal contents may emerge either through the wound or at the drain site, or they may collect near the anastomosis, causing fever, abscess, septicemia, metabolic disturbance, and/or multiple organ failure.
Postoperative hemorrhage	Blood loss within 72 h after surgery, which requires a transfusion of blood
Gastrointestinal bleed	Gastrointestinal bleed is defined as unambiguous clinical or endoscopic evidence of blood in the gastrointestinal tract. Upper gastrointestinal bleeding (or hemorrhage) is that originating proximal to the ligament of Treitz, in practice from the esophagus, stomach, and duodenum. Lower gastrointestinal bleeding is that originating from the small bowel or colon.
Urinary tract infection	Positive urine culture of ≥105 colony forming units/ml with no more than two species of microorganisms, and with at least one sign (among fever > 38 °C, dysuria, suprapubic tenderness, costovertebral angle pain, or tenderness with no other recognized cause.
Acute kidney injury	1.5 times baseline value within 7 days.
Respiratory failure	Postoperative PaO_2_ < 8 kPa (60 mm Hg) on room air, a PaO_2_:FI0_2_ ratio < 40 kPa (300 mmHg) or arterial oxyhemoglobin saturation measured with pulse oximetry < 90 % and requiring oxygen therapy.
Pneumonia	New or progressive and persistent infiltrates or consolidation or cavitation in at least one chest radiograph, with at least one (among fever > 38 °C, white cell count > 12 x 10^9^/l or < 4 x 10^9^/l, altered mental status with no other recognized cause for adults > 70 years old), and at least two (among new onset or change in character in sputum, new onset or worsening cough or dyspnea or tachypnea, rales breath sounds, or worsening gas exchange).
Pulmonary embolism	Pulmonary embolism confirmed by cardiothoracic angiography in the postoperative period.
Acute respiratory distress syndrome	New worsening respiratory symptoms, bilateral opacities in chest imaging, without cardiac failure, and PaO_2_/FiO_2_ < 300 mm Hg.
Cardiogenic pulmonary edema	Evidence of fluid accumulation in the alveoli due to poor cardiac function.
Myocardial infarction	Increase in troponin Ic, with at least one value above the 99^th^ percentile (≥0.04 ng/ml) upper reference limit, and at least one the following criteria: ST or T wave ECG changes or new left bundle branch block, development of pathological Q waves on ECG, echocardiographic evidence of new loss of viable myocardium or new regional wall motion abnormality, or identification of an intracoronary thrombus at angiography.
Myocardial injury	Peak troponin Ic ≥ 0.04 ng/ml (99^th^ percentile).
Arrhythmia	Evidence of cardiac rhythm disturbance in electrocardiograph.
Cardiac arrest	Cessation of cardiac mechanical activity, as confirmed by the absence of signs of circulation.
Stroke	Embolic, thrombotic, or hemorrhagic cerebral event, with persistent residual motor, sensory, or cognitive dysfunction.

The secondary outcomes are the total number of postoperative complications, the real length of hospital stays, and the postoperative mortality.

### Blinding

A coding list has been generated using the SAS software package V.9.4. During surgery and postoperative care, both surgeons and patients are blinded to the allocated group. The material used in the photoplethysmography group and in the control group is similar, in keeping with the blinded design. Therefore, patients in each group remain indistinguishable. Only the anesthesiology staff and the research staff can view the monitor (blinded in the control group) and know the group allocation. The surgeons are the postoperative care providers, who will decide the length of hospital stay while staying totally blinded to the group allocation. The two independent experts are also blinded to the group allocation.

### Intention-to-treat analysis

Patients with a serious undesirable event during the surgery, which justifies invasive hemodynamic monitoring (arterial catheter or cardiac output monitoring), or without photoplethysmography signal will not be treated using the study group algorithm allocation. They will then receive different monitoring or treatment at the discretion of the attending anesthesiologist but will be analyzed according to their initial assigned group following the intention-to-treat principle.

### Sample size estimation

Based on previous published studies [16, 17], two groups of 80 patients are needed to detect a decrease in the incidence from 40 % to 20 % of patients suffering at least one postoperative complication, using a two-sided α-risk at 0.05 and a β-risk at 0.20. Because the incidence of complications is uncertain, checking the number of patients having at least one postoperative complication in the control group will be conducted after 80 patients have been included to verify the power of the study. However, no interim analysis is planned. We anticipate no missing data for the primary outcome (incidence of at least one postoperative complication). For the per-protocol analysis (sensitivity analysis), the handling of missing data (surgery cancelled, failure of photoplethysmography monitoring, or serious undesirable event during the surgery) will be anticipated, with five supplemental patients being included for each group. Nevertheless, all randomized patients will be analyzed in the allocated group for the main intention-to-treat analysis. In addition, to comply with the intent-to-treat analysis, missing data for the primary outcome will handled by multiple imputations (PROC MI in SAS) and analyzed in sensitivity analyses using PROC MIANALYZE in SAS V9.4 (SAS institute, Cary, NC, USA).

### Statistical plan

Categorical variables will be described as percentages, and continuous variables will be described as mean (Standard Deviation) or median (interquartile range), as appropriate. The analysis for the primary outcome will follow the intent-to-treat principle in which all the randomized patients will be analyzed in the assigned group. The principal comparison will be performed by the Fisher exact or the Pearson chi-square test for heterogeneity for the rate of postoperative complications, including the group as independent variables. All statistical analysis will be conducted with SAS V9.4 (SAS institute, Cary, NC). A *p* < 0.05 will denote statistical significance.

### Registration

Data will be collected and registered using electronic case report forms (eCRFs) by a dedicated local research technician. A research coordinator will centralize and verify the data.

### Data collected and registered

Baseline characteristics and pre-randomization data will be collected: sex, age, height, weight, ideal body weight, Lee score [18], smoking status, history and type of diabetes mellitus, dyslipidemia, history of cardiovascular disease (systemic hypertension, ischemic heart disease, valvular heart disease, peripheral vascular disease, or cardiac medications), history of respiratory disease (asthma with chronic treatment, chronic obstructive pulmonary disease requiring corticosteroid daily or oxygen at home or history of hospitalization for decompensation or noninvasive ventilation), history of hepatic disease (Child Pugh classification of cirrhosis [19]), renal insufficiency (classified according to the glomerular filtration rate [20]), history of neoplasia, and baseline bloods (creatinine, bilirubin, albumin, and rate of prothrombin).

During the anesthesia and surgical procedures, the following will be recorded: the type of procedure (first or reoperation), surgical site (right, left, or total colectomy, protectomy, or coloprotectomy), duration of anesthesia and surgery, blood loss and transfusion requirements, all drugs used during anesthesia (anesthetics, opiates, and neuromuscular blocking agents), all administered fluids (number of titrated fluid loadings, and total fluid loading), and all vasoactive drugs.

Data from the EV 1000 monitor will be extracted (mean MAP, mean stroke volume, mean stroke volume variation, mean heart rate, mean MAP/Heart rate ratio, and proportion (%) of time during MAP < 65 mm Hg and < 55 mm Hg). The clinical tolerance of the sensor will be evaluated with a numerical pain scale, and a skin examination (paresthesia, erythema, and necrosis).

During postoperative days 1, 3, and 5, blood samples will be sent for the following analyses: creatinine, troponin Ic, leukocyte, and hemoglobin. If the core temperature > 38 °C, a blood culture, urine culture, and drain fluid culture (if available) will be performed.

From postoperative day 0 until 30 days following surgery, the postoperative complications will be recorded (Table 1).

The real hospital length of stay and the survival status at day 30 following the inclusion will be recorded.

### Record keeping

Consent forms and eCRFs will be retained for 15 years at the University Hospital of Caen in accordance with the French law.

### Study organization

The study promotion is performed by the University Hospital of Caen, France. Industry does not provide financial support; nor is it involved in the study protocol.

### Duration and timeline

Patients from the French University Hospitals of Caen can be included during a 2-year period beginning in December 2014.

Protocol, approval from the ethical committee, financial support, the eCRF, and the interactive web response system (IWRS) were developed in 2014. Inclusions of patients were planned to take place during 2015 and 2016. The database should be closed during 2016 and will be followed by data analysis, manuscript writing, and submission for publication.

## Discussion

The photoplethysmography is a plug-and-play hemodynamic monitoring device, which could allow hemodynamic optimization during the perioperative period for a wide patient population, including patients at moderate surgical risk. The PANEX3 trial is the first randomized controlled study powered to investigate the photoplethysmography as a noninvasive hemodynamic tool in patients scheduled for colorectal surgery compared with the conventional hemodynamic algorithm using mean arterial pressure.

The primary endpoint of the trial is the incidence of at least one postoperative complication. Two independent experts validate the presence of the complication using an a priori classification in accordance with recent guidelines [15]. These experts are not involved in the study and do not work at the same university hospital. They are also blinded to the group allocation. These procedures reinforce the internal validity of the present study. The objective to show a decrease of 50 % in the main goal could be challenging, but this result is in accordance with previous reports [16, 17] and can be considered clinically relevant. If the photoplethysmography algorithm is easy, safe, and effective to use, then this technology could be integrated to the concept of perioperative surgical home [21]. Moreover, another study was designed alongside the PANEX3 trial, and it is investigating the medico-economic aspect of photoplethysmography.

The present study propose a hemodynamic algorithm with photoplethysmography using both stroke volume maximization and continuous mean arterial pressure monitoring compared with a control algorithm using only intermittent mean arterial pressure measurements. Recently, a large retrospective study has described the possible relation between value and duration of arterial hypotension during anesthesia and poor clinical outcome [22]. An algorithm with continuous monitoring of arterial pressure could decrease the duration of hypotension in comparison with conventional intermittent arterial pressure measurement [23], but no outcome study is available with a hemodynamic algorithm using continuous arterial pressure monitoring. The PANEX3 study, which uses a complete and continuous hemodynamic algorithm (mean arterial pressure and stroke volume), is designed to evaluate this strategy.

The limitations of the study require some comment. First, the study population was restricted to colorectal surgery in patients with an intermediate surgical risk. However, the length of surgery and postsurgical recovery time can be long, explaining a possible key role of hemodynamic optimization during this period. Further studies could be developed in other type of surgeries with intermediate surgical risk. Second, the study population was not selected as high-risk surgery because the photoplethysmography hemodynamic monitoring is considered of benefit for the intermediate surgical risk population [4]. However, this population of patients is the more frequently encountered population in practice. Inclusion criteria were large for the study population, reinforcing the external validity of the study.

In conclusion, PANEX3 is a controlled randomized trial powered to test the hypothesis that perioperative hemodynamic optimization using photoplethysmography could decrease the incidence of at least one postoperative complication. The PANEX3 trial also evaluates the impact of a hemodynamic algorithm using photoplethysmography on the total number of postoperative complications, the real length of the hospital stay, and the postoperative mortality.

### Trial status

The trial is ongoing and actively enrolling.

## Abbreviations

eCRF: electronic case report form
IRB: institutional review board
IWRS: interactive web response system
MAP: mean arterial pressure
